# *ARMC10* regulates mitochondrial dynamics and affects mitochondrial function via the Wnt/β‐catenin signalling pathway involved in ischaemic stroke

**DOI:** 10.1111/jcmm.18449

**Published:** 2024-06-25

**Authors:** Yanyang Huang, Zhaojing Zhang, Yatian Xu, Yue Peng, Ruochen Xu, Yingying Luan, Xiaoshuai Bie, Jing Jia, Chi Zhang, Tianyi Han, Baixue Zhou, Zhihao Li, Hong Zheng, Dongzhi Yang, Ying He

**Affiliations:** ^1^ Department of Medical Genetics and Cell Biology, School of Basic Medical Sciences Zhengzhou University Zhengzhou China; ^2^ Reproduction Center The Third Affiliated Hospital of ZhengZhou University Zhengzhou China; ^3^ Clinical Laboratory Center Qingyuan Maternal and Child Health Hospital Qingyuan China; ^4^ School of Life Sciences Zhengzhou University Zhengzhou China

**Keywords:** apoptosis, *ARMC10*, ischaemic stroke, mitochondrial dynamics, Wnt/β‐catenin

## Abstract

Mitochondrial dynamics has emerged as an important target for neuronal protection after cerebral ischaemia/reperfusion. Therefore, the aim of this study was to investigate the mechanism by which *ARMC10* regulation of mitochondrial dynamics affects mitochondrial function involved in ischaemic stroke (IS). Mitochondrial morphology was detected by laser scanning confocal microscopy (LSCM), and mitochondrial ultrastructural alterations were detected by electron microscopy. The expression of mitochondrial dynamics‐related genes *Drp1, Mfn1, Mfn2, Fis1, OPA1* and *ARMC10* and downstream target genes *c‐Myc*, *CyclinD1* and *AXIN2* was detected by RT‐qPCR. Western blot was used to detect the protein expression of β‐catenin, GSK‐3β, p‐GSK‐3β, Bcl‐2 and Bax. DCFH‐DA fluorescent probe was to detect the effect of *ARMC10* on mitochondrial ROS level, Annexin V‐FITC fluorescent probe was to detect the effect of *ARMC10* on apoptosis, and ATP assay kit was to detect the effect of *ARMC10* on ATP production. Mitochondrial dynamics was dysregulated in clinical IS samples and in the OGD/R cell model, and the relative expression of *ARMC10* gene was significantly decreased in IS group (*p* < 0.05). Knockdown and overexpression of *ARMC10* could affect mitochondrial dynamics, mitochondrial function and neuronal apoptosis. Agonist and inhibitor affected mitochondrial function and neuronal apoptosis by targeting Wnt/β‐Catenin signal pathway. In the OGD/R model, *ARMC10* affected mitochondrial function and neuronal apoptosis through the mechanism that regulates Wnt/β‐catenin signalling pathway. *ARMC10* regulates mitochondrial dynamics and protects mitochondrial function by activating Wnt/β‐catenin signalling pathway, to exert neuroprotective effects.

## 1 INTRODUCTION

Ischaemic stroke (IS) is a neurological disease in which insufficient cerebral blood supply due to stenosis or obstruction of cerebral blood supply arteries leads to local ischaemic damage of brain tissues and neurological dysfunction.^1^, ^2^, ^3^ The pathological mechanism of IS is extremely complex, and it is mainly a series of cascading reactions such as oxygen radical damage, calcium overloading, mitochondrial damage, neurotoxicity of excitatory amino acids, inflammatory injury and apoptosis following cerebral ischaemia, which leads to neuronal damage, necrosis and apoptosis, ultimately leading to central nervous system dysfunction.^4^, ^5^, ^6^, ^7^

Mitochondria are the primary site of cellular aerobic respiration and production of reactive oxygen species, and are important mediators of energy homeostasis and cellular signalling.^8^, ^9^, ^10^ The fission and fusion of mitochondria within the cell are known as mitochondrial dynamics and are controlled by a series of proteins.^11^, ^12^ Several genes controlling mitochondrial fission/fusion dynamics have been identified, such as fission protein 1 (*Fis1*), mitochondrial fission factor (*Mff*), dynamic‐related protein 1 (*Drp1*) and mitofusin 1/2 (*Mfn1/2*). Generally, *Drp1* and *Fis1* control mitochondrial fission, while *Mfn1/2*, Optic Atrophy 1(*OPA1*) control mitochondrial fusion. Imbalance in mitochondrial dynamics usually lead to mitochondrial dysfunction and affect energy metabolism and neuronal function after stroke by regulating mitochondrial number, morphology and function.^13^, ^14^, ^15^

In ischaemic stroke, inadequate oxygen and nutrient supply disrupts energy homeostasis and adenosine triphosphate (ATP) synthesis, ultimately leading to mitochondrial dysfunction and permanent brain damage. Many studies have found that mitochondrial dynamics play an important role in ischaemic stroke. For example, Liu et al. noted that hyperglycaemia exacerbates cerebral ischaemia/reperfusion‐induced neuronal injury by activating cellular autophagy and mitochondrial division via ERK1/2.^16^ Wu et al. investigated that ligustilide induces *Drp1*‐mediated mitochondrial fragmentation in vivo and in vitro through activation of the AMPK signalling pathway, protecting against neural injury and preventing ischaemic stroke.^17^ Li et al. expounded that OMA1‐mediated OPA1 cleavage (S1‐OPA1) and then S1‐OPA1 exacerbates neuronal mitochondrial fragmentation and injury and participates in neuronal ischaemia–reperfusion injury in a GTPase‐dependent manner.^18^

The *armadillo repeat containing 10* (*ARMC10*) gene, also known as *splicing variant involved in hepatocarcinogenesis* (*SVH*), is located on chromosome 7 and encodes the ARMC10 (SVH) protein. Different splice variants can form four isoforms of SVH‐A, B, C and D proteins, which can be expressed in a variety of tissues and are particularly highly expressed in human brain and kidney.^19^ Serrat et al. found that *ARMC10* have unique features in the functional regulation of mitochondrial dynamics.^20^ Jan Kriska noted that the Wnt/β‐catenin signalling pathway promotes neurogenesis in neural stem/progenitor cells and activates neuronal differentiation in the subventricular zone.^21^ However, there are few reports on the involvement of *ARMC10* in the pathological process and mechanism of ischaemic stroke. Therefore, the aim of the present study was to investigate the role of *ARMC10* through the altering mitochondrial dynamics involved in the pathological process of ischaemic stroke, thereby affecting neuronal function.

In the present study, we firstly investigated the correlation between mitochondrial dynamics and IS, and then, we constructed *ARMC10* knockdown and overexpression cell models to confirm that *ARMC10* regulates mitochondrial dynamics and thus affects mitochondrial function and neuronal apoptosis. Next, we detected the expression levels of key molecules and downstream target genes of the Wnt/β‐catenin signalling pathway, to explore the mechanism of *ARMC10* regulating mitochondrial function involved in IS neuronal injury. Finally, OGD/R model was constructed in SH‐SY5Y cells to verify the hypothesis.

## 2 MATERIALS AND METHODS

### 2.1 Study population

Fifty‐three patients with first‐ever IS who attended the First People's Hospital of Zhengzhou City from October 2018 to February 2019 were included in this study, while 53 individuals who underwent health examination at the First Affiliated Hospital of Henan University of Traditional Chinese Medicine were randomly selected as controls. The selected patients had to be admitted to the hospital within 2 days after the occurrence of stroke. IS was diagnosed according to the diagnostic criteria revised by the Fourth National Academic Conference on Cerebrovascular Disease. Those with family history of diabetes mellitus, cardiovascular disease and hypertension were excluded. All study subjects were Han Chinese and not related to each other by blood.

The study protocol was approved by the Ethical Committees of Zhengzhou University. All study participants signed an informed consent form.

### 2.2 Cell transfection and oxygen–glucose deprivation/re‐oxygenation (OGD/R) treatments

#### 2.2.1 Cell culture

SH‐SY5Y, a neuroblastoma cell line, Cells were purchased from National Collection of Authenticated Cell Cultures. SH‐SY5Y was cultured with MEM/F‐12 (1:1, Hyclone, China) containing 10% foetal bovine serum (FBS, BI, Israel) and 1% Streptomycin mixture (Solarbio, China). When the cell density reaches more than 80%, it can be subcultured. Washed cell with PBS twice and digested with 1 mL trypsin–EDTA for 2 min. Collected cells after termination of digestion, centrifuge at 1000 rpm for 5 min. Resuspension cell and placed in a 25 T culture flask bottle, culture it in a constant temperature incubator. Culture conditions: 5% CO_2_, 37°C, humidity 95%.

#### 2.2.2 Cell transfection

The overexpression plasmid vector of *ARMC10* constructed by Hippobio (Zhejiang, China). SH‐SY5Y cells were cultured in six‐well plates at a density of 6 × 10^5^ cells per well and transfected with siRNA and overexpression plasmid of *ARMC10* gene, Ribo FECT™ CP (RIBBIO, China) and Lipofectamine™ 3000 Transfection Reagent (ThermoFisher, America) were used in accordance with the manufacturer's protocol. Follow‐up experiments were carried out after 48 h of culture.

#### 2.2.3 Oxygen–glucose deprivation/re‐oxygenation (OGD/R) treatment

SH‐SY5Y cells were seeded in 6‐well plate and perform OGD/R processing when the density reaches 80%. Discarded the culture medium, washed the cells gently with PBS and add sugar‐free DMEM medium (BI, Israel) (serum‐free) and placed in 37°C anaerobic incubator for 24 h. Subsequently, the medium was changed to normal medium and cultured in CO_2_ incubator for 8 h. CCK‐8 Kit (Solarbio, China) was used to detect cell viability of different time points, OGD24 h/R8 h was selected to establish OGD/R model. Each group was provided with 3 wells.

### 2.3 Extraction and purification of DNA and RNA, RNA reverse transcription

#### 2.3.1 Extraction of peripheral blood PBMCs

Transfer blood from EDTA‐Na_2_ tube to 15 mL centrifuge tube, adding equal volume of physiological saline. Mixed with 3 mL of human peripheral blood mononuclear cell isolation solution, centrifuge at 2000 rpm for 25 min. Suck out the leukocytes and transfer to new 15 mL centrifuge tube, five‐fold volume of PBS solution suspend cells, centrifuge at 1000 rpm for 5 min, abandon supernatant and add 2 mL of 1640 complete medium for Mitotracker Red staining.

#### 2.3.2 RNA extraction and RT‐qPCR

Cells were collected and mixed with 1 mL Trizol and left on ice 5 min. Add 0.2 mL chloroform per 1 mL Trizol and place 3 min on ice. Removed upper water phase into new tube after 4°C 12,000×*g* centrifugal 15 min, mixed with 500 μL isopropyl alcohol and set on ice 10 min and centrifuged again. Used 75% ethanol wash subside twice, dry it and add RNase‐free water. Detect RNA by NanoDrop 2000 spectrophotometer. First‐strand cDNA was synthesized using Reverse transcriptase kit (YEASEN, China).

#### 2.3.3 RT‐qPCR

Refer to the Hieff qPCR SYBR Green Master Mix manual (YEASEN, China) to prepare the RT‐qPCR reaction system and set the reaction conditions. The primers were used for RT‐qPCR were synthesized by Tsingke Biotechnology Company (China). Relative RNA levels detected by RT‐qPCR using the SYBR Green method. The relative expression of RNAs was normalized to β‐actin/β‐catenin mRNA calculated using the comparative CT method. Primer sequences were shown in Table 1.

**TABLE 1:** Primer sequences of RT‐qPCR.

Gene	Primers
*ARMC10*	F:5′‐TCTCTGGTCCTCTGAACTCTGCTG‐3′
	R: 5′‐TGTGCTGGTGGTCATTGGTAACAG‐3′
*Drp1*	F: 5′‐GAGATGGTGTTCAAGAACCAAC‐3′
	R: 5′‐CAATAACCTCACAATCTCGCTG‐3′
*Fis1*	F: 5′‐CTTGCTGTGTCCAAGTCCAA‐3′
	R: 5′‐GCTGAAGGACGAATCTCAGG‐3′
*OPA1*	F: 5′‐TCTGCACACTCAGTTGAAGTAT‐3′
	R: 5′‐GCCTTTGTCATCTTTCTGCAAT‐3′
*Mfn1*	F: 5′‐GTGGCAAACAAAGTTTCATGTG‐3′
	R: 5′‐CACTAAGGCGTTTACTTCATCG‐3′
*Mfn2*	F: 5′‐GTGCTTCTCCCTCAACTATGAC‐3′
	R: 5′‐ATCCGAGAGAGAAATGGAACTC‐3′
*β‐actin*	F: 5′‐CCTGGCACCCAGCACAAT‐3′
	R: 5′‐GGGCCGGACTCGTCATAC‐3′
*c‐Myc*	F: 5′‐CGACGAGACCTTCATCAAAAAC‐3′
	R: 5′‐CTTCTCTGAGACGAGCTTGG‐3′
*CyclinD1*	F: 5′‐GTCCTACTTCAAATGTGTGCAG‐3′
	R: 5′‐GGGATGGTCTCCTTCATCTTAG‐3
*AXIN2*	F: 5′‐CTCCGAGCTCACACTCAATTC‐3′
	R: 5′‐GACAGGTGATCGTCCAGTATC‐3′
*β‐catenin*	F: 5′‐TGGATTGATTCGAAATCTTGCC‐3′
	R: 5′‐GAACAAGCAACTGAACTAGTCG‐3′

### 2.4 Protein extraction and western blot

#### 2.4.1 Nuclear/cytoplasmic separation protein extraction

Cytoplasmic protein extraction reagents A and B were added, respectively, vortex to fully disperse the cell precipitation, ice bath. Centrifugation at 12,000–16,000×*g* for 5 min at 4°C, the supernatant obtained was cytoplasmic protein, transferred to a new EP tube and temporarily placed on ice. Cytoplasmic protein extraction reagent was added into precipitate, vortex 15 s per 15 min, centrifuged again after 40 min. Remove supernatant into new tube, that is, the nuclear protein. Total protein was extracted with RIPA buffer(high) (Solarbio, China) and PMSF (Solarbio, China) using BCA protein concentration determination kit (Solarbio, China) determine the concentration of total protein. Boil samples 10 min and immediately put on the ice.

According to the molecular weight of the target protein, prepared PAGE Gel (Epizyme Biotech, China). Fully separate protein through electrophoresis, then transferred onto PVDF membranes and blocked with 5% skim milk powder. The following protein level were detected by western blot: ARMC10, Cyclin D1, AXIN2, cMyc, Bcl‐2, Bax, p‐GSK‐3β, GSK‐3β, β‐Catenin, ACTB, Lamin B1, Beta Tubulin and GAPDH.

Antibodies of incubation include ARMC10 Rabbit Polyclonal Antibody (Proteintech, China), Cyclin D1 Antibody (Abmart, China), Axin2 Rabbit Antibody (Abmart, China), cMyc Rabbit Antibody (Abmart, China), Bcl‐2 Rabbit Antibody (Abmart, China), Bax Rabbit Antibody (Cell Signaling Technology, American), p‐GSK‐3β (Ser9) XP® Rabbit mAb (Cell Signaling Technology, American), GSK‐3β (D5C5Z) XP® Rabbit mAb (Cell Signaling Technology, American), β‐Catenin (D10A8) XP® Rabbit mAb (Cell Signaling Technology, American), ACTB Rabbit mAb (High Dilution) (ABclonal, China), Lamin B1 (Cell Signaling Technology, American), Beta Tubulin Rabbit Polyclonal antibody (Proteintech, China) and Anti‐Rabbit IgG (Proteintech, China). Image information was collected with ultra‐sensitive exposure system.

### 2.5 Detection of mitochondrial biological function and apoptosis assays

#### 2.5.1 ATP assay

Mitochondrial ATP content was detected using ATP Assay Kit (Beyotime Biotechnology, China). Remove culture medium and add 200 μL lysis buffer, vortex to lyse fully, centrifuged with cryogenic centrifuge at 12,000×*g* for 5 min. Take the supernatant for future experiment. Add 100 μL ATP detection working fluid to the detection hole and place 5 min at room temperature. Add 50 μL standard solution or sample in per hole then luminescence was detected with CentroLB960 Micro‐orifice plate luminescence detector.

#### 2.5.2 Reactive oxygen species assay

Intracellular ROS was measured by means of DCFH‐DA fluorescent probe (Reactive Oxygen Species Assay Kit, Beyotime Biotechnology, China). Cells were collected and washed with PBS and added 1 mL diluted DCFH‐DA; positive control group was added with 1 mL diluted group. After incubate cells in 37°C incubator 20 min, wash cells with PBS three times and detect DCF on the flow cytometry (BD, America). Group was added to the positive control wells.

#### 2.5.3 Apoptosis assays

Apoptosis was evaluated using the Annexin V‐FITC/PI Apoptosis Detection kit(Beyotime Biotechnology, China).Collected cells and washed with PBS, and resuspended in 500 μL binding buffer. 10 × 10^4^ suspended cells were centrifuged by 1300 rpm for 5 min, the supernatant was discarded and mixed with 195 μL Annexin V‐FITC binding solution. Add 5 μL Annexin V‐FITC and 10 μL propidium iodide (PI) staining solution in turn and mix gently. Incubate 20 min without light at room temperature and shake every other 5 min during the period to improve dyeing efficiency. Cold storage away from light, flow detection was completed within 1 h.

### 2.6 Statistical analysis

The images of western blot were processed by ImageJ software, and the experimental data were statistically analysed by GraphPad Prism 8.0.1. All the experimental data were tested for normality and homogeneity of variance, and then, independent sample *t*‐test or one‐way analysis of variance (one‐way ANOVA) was performed. The statistical results were expressed in the form of mean ± standard deviation (mean ± SD), *α* = 0.05, *p* < 0.05 indicated that the difference was statistically significant.

## 3 RESULTS

### 3.1 Changes of mitochondrial dynamics and function in clinical samples and OGD models

**FIGURE 1:**
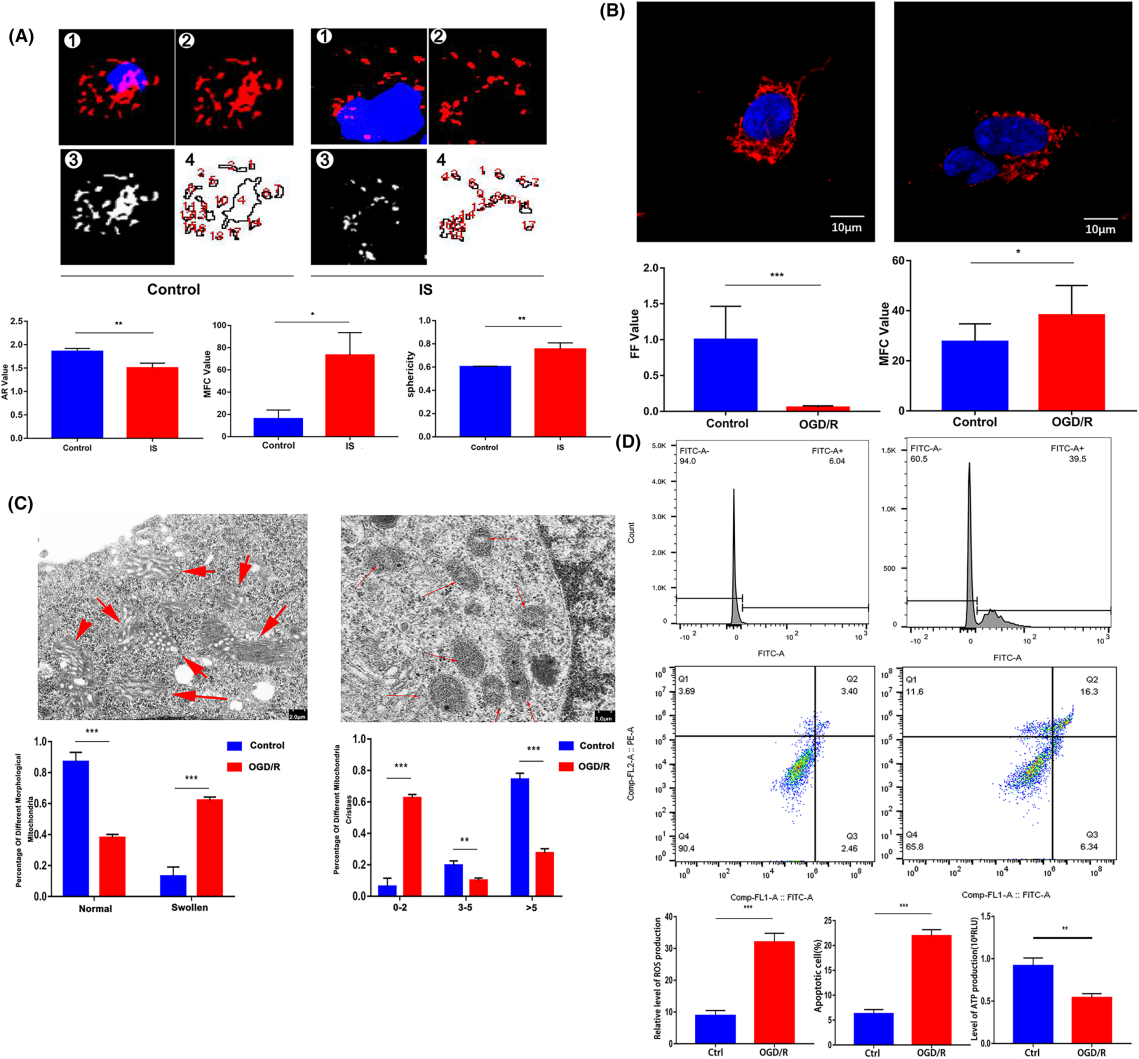
Changes of mitochondrial dynamics and function in clinical samples and OGD models (*p < 0.05, **p < 0.01, ***p < 0.001). (A) The mitochondrial morphology of PBMCs was compared between IS group and control group with LSCM (Mitotracker Red was used for mitochondrial staining).(B) Comparison of mitochondrial morphology between control group and OGD/R group (Mitotracker Red was used for mitochondrial staining). (C) The changes of mitochondrial ultrastructure after OGD/R treatment were observed by electron microscopy. (D) After OGD/R, flow cytometry was used to detect the level of ROS and apoptosis, ATP detection with Microplate Luminometer.

To explore the correlation between ischaemic stroke and mitochondrial dynamics, the peripheral blood of three IS patients and three healthy controls were collected to extract mononuclear cells, and then, the morphological characteristics of mitochondria were observed with LSCM. The results showed significant fragmentation of mitochondria in IS group. The mitochondrial aspect ratio (AR) value was significantly lower than that in control, while the mitochondrial fragmentation count (MFC) and sphericity results were opposite to the AR trend. (Figure 1A).

The in vitro experimental results also showed that OGD/R treatment had an impact on mitochondrial morphology. The value of form factor (FF) in OGD/R group was significantly lower, while the MFC value of the OGD/R group was significantly higher than control (Figure 1B). After OGD/R, the ultrastructure of mitochondria underwent significant changes, with a significant increase in the proportion of swollen mitochondria and a significant decrease in the proportion of mitochondria with more than 5 cristae (Figure 1C).

Mitochondrial dysfunction occurs after OGD/R treatment, characterized by an increase in mitochondrial ROS levels and a decrease in ATP production. In addition, the apoptosis rate of SHSY‐5Y cells in the OGD/R group significantly increased, indicating that OGD/R treatment increased neuronal apoptosis (Figure 1D).

### 3.2 Exploring genes that regulate mitochondrial dynamics changes

**FIGURE 2:**
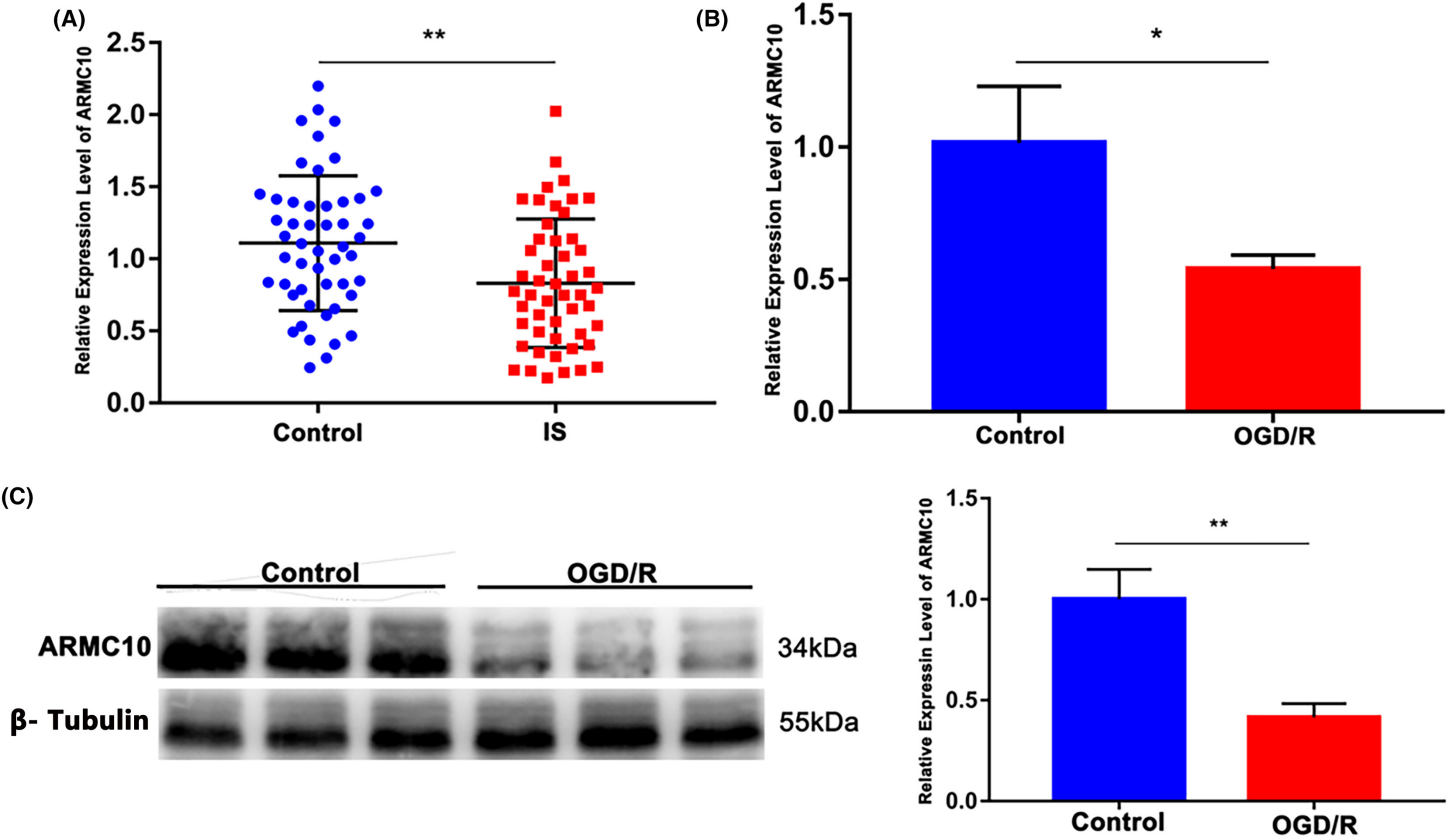
Exploring genes that regulate mitochondrial dynamics changes. (*p < 0.05, **p < 0.01, ***p < 0.001). (A) RT‐qPCR detect the expression of *ARMC10* in peripheral blood of the control group and the IS group. (B) RT‐qPCR detect *ARMC10* expression levels of SH‐SY5Y in control group and OGD/R group. (C) *ARMC10* expression levels of SH‐SY5Y in ODG/R group and control group.

In order to further explore the genes that regulate mitochondrial dynamics changes, RT‐qPCR was used to detect the expression of mitochondrial dynamics‐related genes in the IS group and the control group. The results showed that the relative expression level of *ARMC10* in IS group was significantly lower than that in normal control (*p* < 0.01), but there was no significant difference in the expression level of *Drp1, Mfn1, Mfn2, Fis1 and OPA1* between the two groups (*p* > 0.05) (Figure S1A). Consistent with the clinical samples, the results of RT‐qPCR and western blot showed that *ARMC10* expression is significantly reduced after ischaemia–reperfusion (I/R) injury (Figure 2B,C; Figure S1B).

### 3.3 Verify the effect of *ARMC10* on mitochondrial dynamics

**FIGURE 3:**
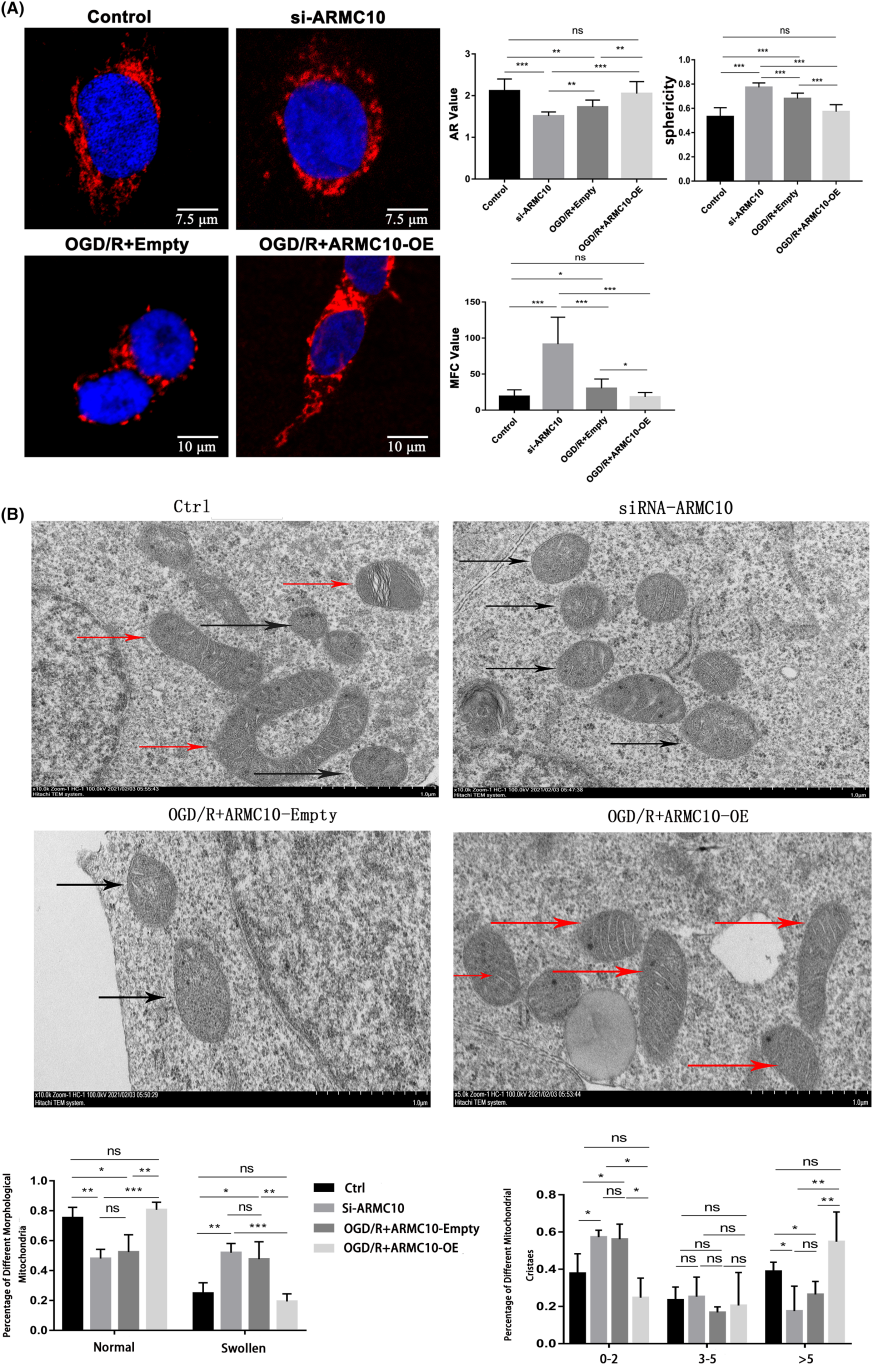
Verify the effect of *ARMC10* on mitochondrial dynamics (*p < 0.05, **p < 0.01, ***p < 0.001). (A) Effect of *ARMC10* expression on mitochondrial morphology was observed by LSCM (Mitotracker Red was used for mitochondrial staining). (B) The effect of *ARMC10* expression on mitochondrial ultrastructure was observed by electron microscopy.

Mitochondrial morphology analysis showed that knocking down *ARMC10* resulted in significant fragmentation of mitochondrial morphology. The AR value significantly decreased, while the sphericity and MFC value significantly increased. While overexpression of *ARMC10* could reduce mitochondrial disruption caused by OGD/R (Figure 3A). In addition, the ultrastructure of mitochondria also changed significantly after knockdown of *ARMC10*, showing that the proportion of swollen mitochondria increased significantly and the number of crista decreased significantly. Compared with OGD/R + *ARMC10* Empty, the proportion of mitochondrial swelling was significantly reduced, and the number of crista was significantly increased after overexpression of *ARMC10* (Figure 3B).

### 3.4 *ARMC10* affects mitochondrial function and Neuronal apoptosis

**FIGURE 4:**
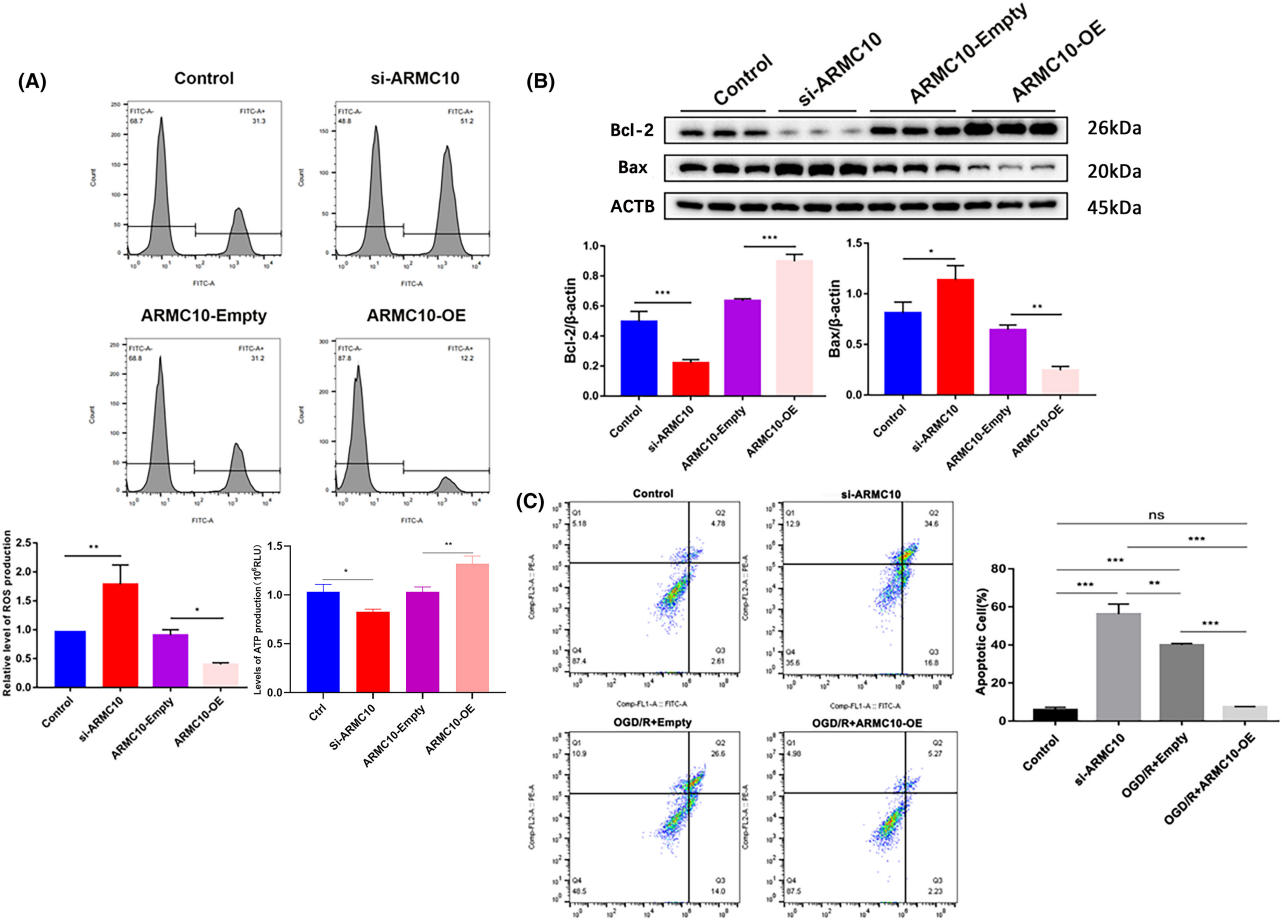
*ARMC10* affects mitochondrial function and Neuronal apoptosis (*p < 0.05, **p < 0.01, ***p < 0.001). (A) Flow cytometry detect the effect of *ARMC10* expression on mitochondrial function. (B) Western blot detect the effect of *ARMC10* expression on apoptosis proteins. (C) Flow cytometry was used to detect the effect of *ARMC10* expression on cell apoptosis.

Then, we validated that the change of *ARMC10* expression level affected mitochondrial function and neuronal apoptosis. Transfection of si‐*ARMC10* significantly increased ROS production and decreased ATP production, while overexpression of *ARMC10* significantly decreased ROS content and increased ATP production (Figure 4A). In addition, silencing *ARMC10* could up‐regulate the expression of pro‐apoptotic protein Bax and reduce the level of anti‐apoptotic protein Bcl‐2, while overexpression of *ARMC10* could lead to the opposite result (Figure 4B). The results of flow cytometry were consistent with western blot, indicating a significant rise in cell apoptosis in si‐*ARMC10* group. The cell apoptosis rate of OGD/R + Empty plasmid group was significantly higher, and overexpression of *ARMC10* could rescue OGD/R induced cell apoptosis (Figure 4C).

### 3.5 *ARMC10* activates Wnt/β‐Catenin signal pathway and initiates the expression of downstream target genes

**FIGURE 5:**
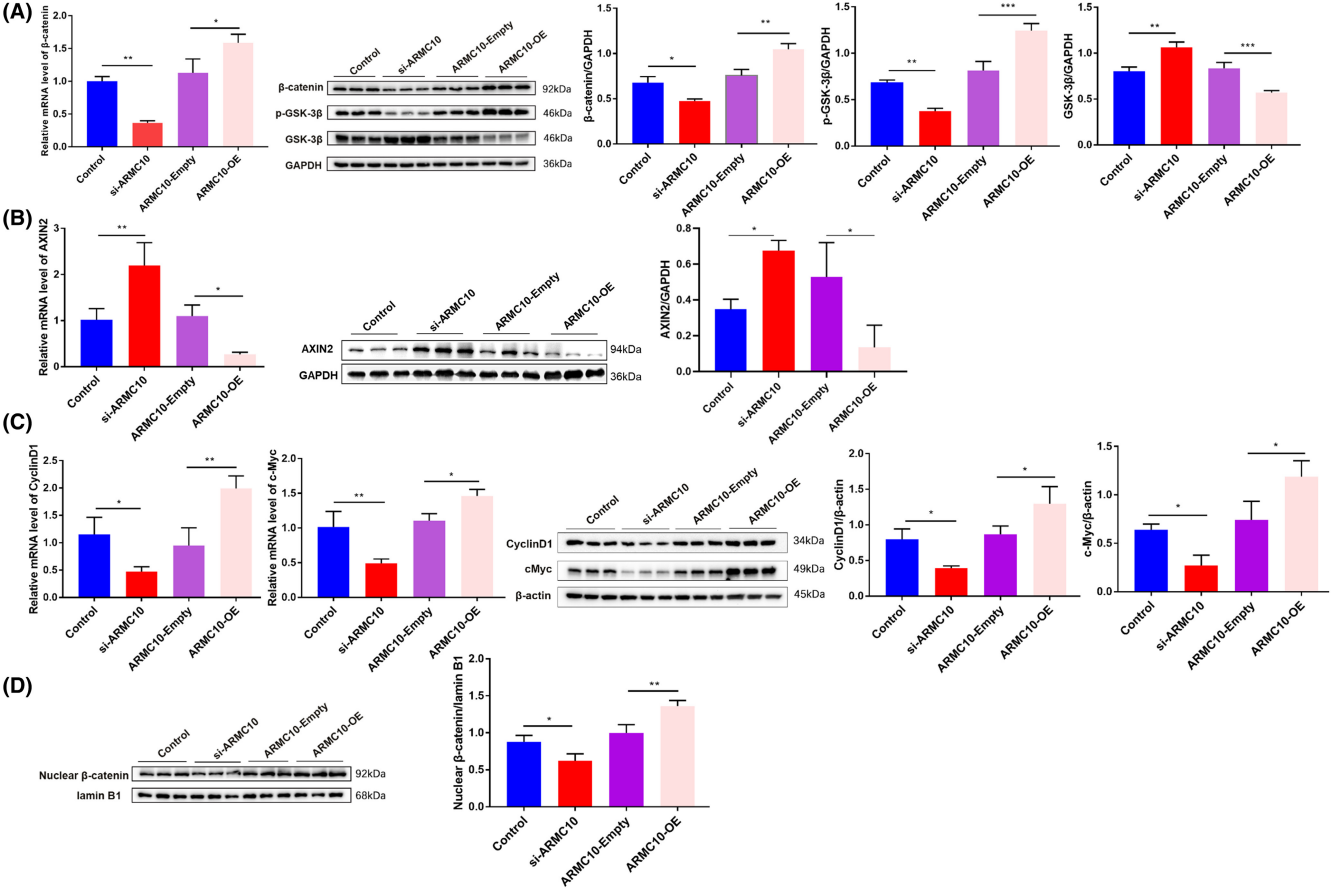
*ARMC10* activates Wnt/β‐Catenin signal pathway and initiates the expression of downstream target genes (*p < 0.05, **p < 0.01, ***p < 0.001). (A) RT‐qPCR and western blot detected protein expression levels of key molecules in Wnt/β‐catenin signalling pathway. (B) RT‐qPCR and western blot detected expression of *AXIN2*. (C) RT‐qPCR and western blot to determine the changes of downstream target genes of Wnt/β‐catenin signalling pathway under different conditions. (D) The level of β‐catenin protein in the nucleus was detected by nucleoplasm separation.

Next, we explored whether *ARMC10* activate Wnt/β‐Catenin signalling pathway by which *ARMC10* affects mitochondrial function and leads to neuronal apoptosis. In SH‐SY5Y cells, the expression levels of *ARMC10* were altered by transfection of si‐*ARMC10*, *ARMC10* overexpression plasmids and pCDH empty plasmids, respectively. We hypothesized that knockdown of *ARMC10* affected key proteins of Wnt/β‐catenin signal pathway, including β‐catenin, GSK‐3β and p‐GSK‐3β, which have been reported.^22^, ^23^ Western blot and RT‐qPCR confirmed that the expression of β‐catenin and p‐GSK‐3β decreased significantly, while the expression level of GSK‐3β increased significantly. In addition, the results of western blot and RT‐qPCR showed that the overexpression of *ARMC10* up‐regulated the level of β‐catenin and p‐GSK‐3β, and the expression of GSK‐3β was reduced at the same time (Figure 5A; Figure S2A).

The transfer level of β‐catenin from cytoplasm to nucleus is crucial step that Wnt/β‐catenin signalling pathway plays an important role in biological functions. Therefore, SH‐SY5Y cells were collected for nucleocytoplasmic separation and extraction of nuclear protein, and then, the transfer level of β‐catenin protein from cytoplasm to nucleus was detected by western blot. After knocking down *ARMC10*, the amount of β‐catenin transferred from cytoplasm to nucleus decreased significantly, while after overexpression of *ARMC10*, the decrease of β‐catenin transformation was rescued (Figure 5D).

After Wnt/ β‐catenin signalling pathway was activated, β‐catenin transferred from cytoplasm to nucleus, initiating transcription and translation of downstream target genes.^24^, ^25^ Knocking down *ARMC10* significantly up‐regulated the expression of *AXIN2* at both mRNA and protein level, while the expression of *AXIN2* decreased significantly after overexpression of *ARMC10* (Figure 5B). The expression of *c‐Myc* and *CyclinD1* significantly decreased in si‐*ARMC10* group and up‐regulated the expression of *c‐Myc* and *CyclinD1* after overexpression of *ARMC10* (Figure 5C).

### 3.6 Agonist and inhibitor affect mitochondrial function and neuronal apoptosis by targeting Wnt/β‐catenin signal pathway

**FIGURE 6:**
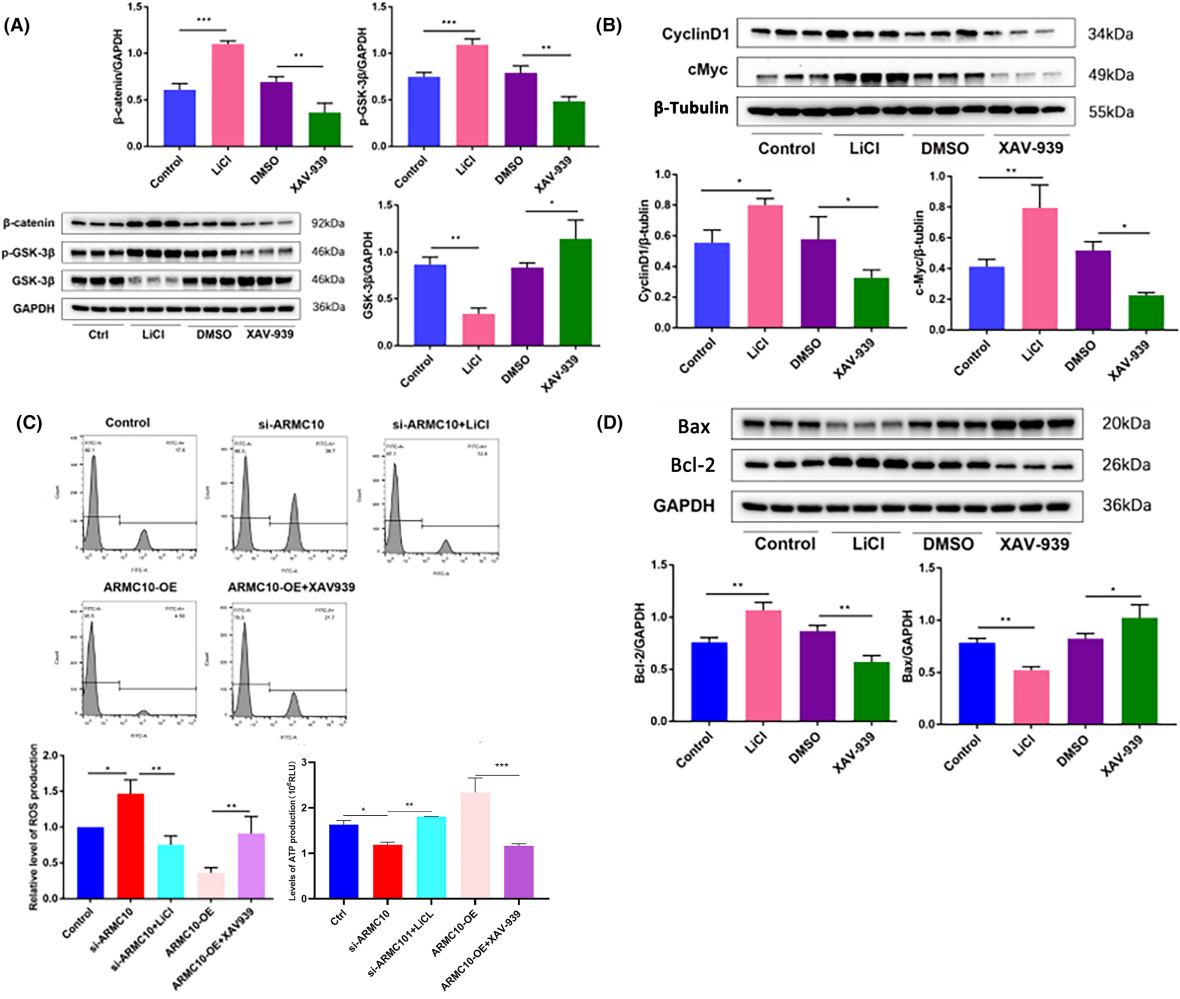
Agonist and inhibitor affect mitochondrial function and neuronal apoptosis by targeting Wnt/β‐Catenin signal pathway (*p < 0.05, **p < 0.01, ***p < 0.001). (A) Western blot detected protein expression levels of key molecules in Wnt/β‐catenin signalling pathway. (B) Western blot was used to detect the expression of downstream target genes of Wnt/β‐catenin signalling pathway. (C) FCM was used to detect effects of LiCl (20 mM) and XAV‐939 (10 μM) on mitochondrial function. (D) Western blot was used to detect the effect of ARMC10 expression on apoptosis proteins.

LiCl and XAV‐939 are commonly used agonist and inhibitor targeting the Wnt/β‐catenin signal pathway. Therefore, LiCl and XAV‐939 were used to detect their effects on mitochondrial function and neuronal apoptosis. Protein expression levels of β‐catenin, GSK‐3β and p‐GSK‐3β were detected by western blot, and the results showed that LiCl treatment significantly up‐regulated the protein expression of β‐catenin and p‐GSK‐3β and inhibited the expression of GSK‐3β; compared with DMSO, the protein expression of β‐catenin and p‐GSK‐3β was down‐regulated and the expression of GSK‐3β was significantly increased in XAV‐939 group (Figure 6A; Figure S2B). Expression level of *CyclinD1* and *c‐Myc* increased significantly by treatment with LiCl, XAV‐939 could down‐regulate the level of *CyclinD1* and *c‐Myc* (Figure 6B). The application of LiCl attenuated the increasing of ROS production and decreasing of ATP production induced by *ARMC10* silencing. The use of pathway inhibitors showed the same effect (Figure 6C). After LiCl treatment, the expression level of pro‐apoptotic protein Bax was significantly down‐regulated, and the expression level of anti‐apoptotic protein Bcl‐2 was significantly up‐regulated, and opposite results after the treatment of XAV‐939 were observed (Figure 6D).

### 3.7 In OGD/R model, *ARMC10* regulates Wnt/β‐catenin signalling pathway affecting mitochondrial function and neuronal apoptosis

**FIGURE 7:**
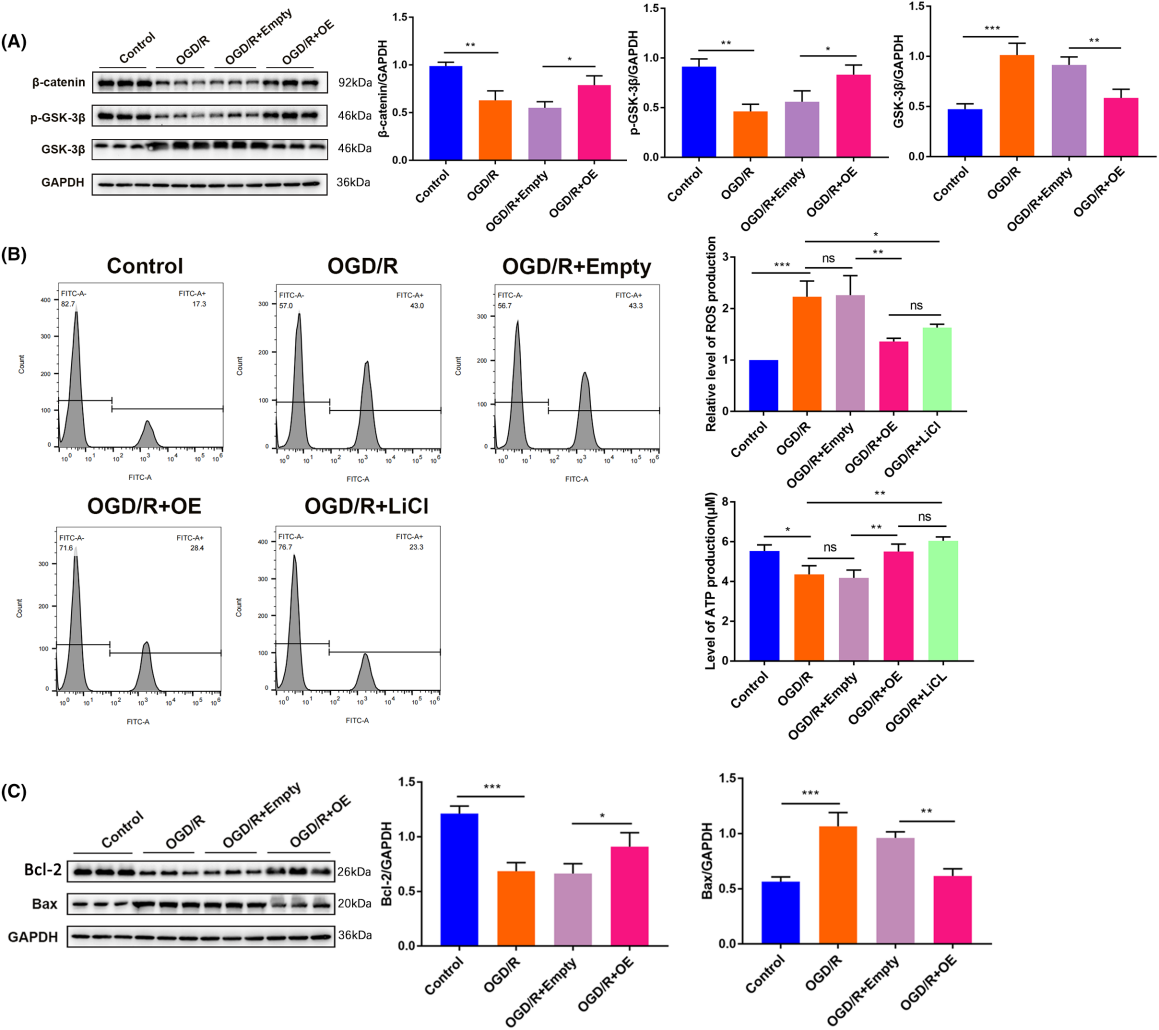
In OGD/R model, *ARMC10* regulates Wnt/β‐Catenin signalling pathway affecting mitochondrial function and neuronal apoptosis (*p < 0.05, **p < 0.01, ***p < 0.001). (A) The key molecules of Wnt/β‐catenin signalling pathway were detected by western blot. (B) FCM was used to detect the function of different groups of mitochondria. (C) Expression of apoptosis‐related proteins under different conditions. (A and C shares the same batch of GAPDH bands in their WB results)

Finally, we validated that *ARMC10* activates Wnt/β‐catenin pathway and affects mitochondrial function and neuronal apoptosis in OGD/R cell model. Under the condition of OGD/R, the expression of β‐catenin and p‐GSK‐3β decreased significantly, while the expression of GSK‐3β increased significantly, and the overexpression of *ARCM10* rescued the down‐regulation of β‐catenin and p‐GSK‐3β and the up‐regulation of GSK‐3β induced by OGD/R (Figure 7A; Figure S2C). The results of RT‐qPCR and western blot showed that β‐catenin nuclear translocation was reduced and AXIN2 expression was increased, while cMyc and CyclinD1 expression was decreased after OGD/R treatment, but the above effects were reversed when *ARMC10* was overexpressed (Figure S3).

Compared with the control group, the OGD/R group showed a significantly increasing in ROS generation and a significantly decreasing in the level of ATP generation. Relative to the OGD/R + Empty group, OGD/R + OE group showed that ROS production was significantly decreased and ATP production level was significantly increased; OGD/R + LiCl group showed a significantly inhibited in ROS production and a significantly rise in ATP production level compared with OGD/R group (Figure 7B).

The expression of apoptotic protein Bax protein was significantly up‐regulated and the expression of anti‐apoptotic protein Bcl‐2 protein was significantly down‐regulated in the OGD/R group compared with the control group; the expression of Bax protein was significantly down‐regulated and the expression of Bcl‐2 protein was significantly up‐regulated in the OGD/R + ARMC10‐OE group relative to the OGD/R + Empty group. The results are shown in Figure 7C.

## 4 DISCUSSION

Mitochondrial dynamics, a key mechanism for mitochondrial quality control, has emerged as an important target for neuronal protection after cerebral ischaemia/reperfusion.^26^ We found that mitochondrial dynamics were dysregulated in clinical IS samples and in the OGD/R cell model, mitochondrial morphology of PBMCs from IS patients exhibited significant fragmentation changes. Similarly, we observed mitochondrial fragmentation, mitochondrial swelling and a reduction in cristae in the OGD/R cell model. The ultrastructure of mitochondria changed markedly after OGD/R: the proportion of swollen mitochondria increased markedly, and the proportion of mitochondria with cristae more than five decreased markedly. Zhang et al. investigated the effects of 2‐week exercise preconditioning on mitochondrial dynamics in an adult male rat model of stroke and found that OPA1 was up‐regulated and cerebral oedema was reduced after cerebral ischaemia.^27^ This was consistent with our findings. Ischaemic stroke induces an increase in mitochondrial fission, which contributes to increased mitochondrial energy production. However, excessive fission impairs normal mitochondrial function. Dysfunctional mitochondrial dynamics can impair mitochondrial distribution and transport in neurons, thereby shortening energy supply and inducing apoptosis. Restoring the balance between mitochondrial fusion and fission is beneficial for recovery from ischaemic stroke. Mitochondrial dynamics are recognized as a potential therapeutic target for ischaemic stroke.

So, which genes regulate mitochondrial dynamics and influence mitochondrial function during the pathology of ischaemic stroke? Chen et al. found that in the transient global cerebral ischaemia model of rat, hypoglycaemia and hypoxia can depress the phosphorylation of Drp1 at Ser616 in the CA1 region of the hippocampus, which leads to increased mitochondrial division.^28^ Li et al. constructed a Glucose–Oxygen Deprivation/Reperfusion (OGD/R) model in the PC‐12 cell line and found that the expression of the mitochondrial fusion gene *Mfn2* was reduced, and the mitochondrial morphology was fragmented.^29^ Therefore, several genes which control mitochondrial fission/fusion dynamics such as *Drp1, Mfn1, Mfn2, Fis1, OPA1* and *ARMC10* were selected. The results showed that the relative expression level of *ARMC10* in IS patients was significantly lower than that in normal control (*p* < 0.01), but there was no significant difference in the expression levels of *Drp1, Mfn1, Mfn2, Fis1 and OPA1* between the two groups (*p* > 0.05). Based on literature review, there was little relevant study on the relationship between *ARMC10* and ischaemic stroke currently. *ARMC10*, as a gene associated with mitochondrial dynamics that may be involved in the pathologic process of IS, has attracted our attention and interest.

First of all, we explored whether *ARMC10* affects mitochondria dynamic. Morphology analysis showed that knockdown of *ARMC10* resulted in significant fragmentation of mitochondrial morphology, the AR value was significantly reduced, while the sphericity and MFC value were significantly increased. In addition, overexpression of *ARMC10* reduced mitochondrial disruption. In addition, the ultrastructure of mitochondria also changed significantly after knockdown of *ARMC10*, which was manifested as a significant increase in the proportion of swollen mitochondria and a significant decrease in the number of crista. Overexpression of *ARMC10* resulted in a significant decrease in the proportion of swollen mitochondria and a significant increase in the number of cristae. In conclusion, knockdown of *ARMC10* in SH‐SY5Y cells significantly impaired mitochondrial dynamics, while overexpression of *ARMC10* rescued the impairment of mitochondrial dynamics.

Mitochondrial dynamics is an important mechanism for maintaining mitochondrial homeostasis and is closely related to mitochondrial function.^30^, ^31^ ATP synthesis and ROS production are often used as important indicators for evaluating mitochondrial function.^32^, ^33^ Our results showed that transfection of si‐*ARMC10* significantly increased ROS production and decreased ATP production, whereas overexpression of *ARMC10* significantly decreased ROS content and ATP production. Mitochondrial fusion/fission dynamics broadly affect neuronal function, including neuronal survival and plasticity. Mitochondrial dysfunction can cause apoptosis through the release of Cytochrome‐c (Cyt‐C) or pro‐apoptotic proteins. Our results showed that silencing *ARMC10* could up‐regulate the expression of pro‐apoptotic protein Bax and reduce the level of anti‐apoptotic protein Bcl‐2, while overexpression of *ARMC10* can lead to the opposite result. The cell apoptosis rate of OGD/R + Empty plasmid group was significantly higher, and overexpression of *ARMC10* could rescue OGD/R induced cell apoptosis. Serrat et al. found that ARMC10 protein was localized in the outer mitochondrial membrane by immunofluorescence staining methods and molecular labelling techniques. In addition, an Alzheimer's disease (AD) model was constructed in mouse neuronal cells, and it was found that overexpression of *ARMC10* reduced the number of mitochondria in neuronal cells in the exercise state and promoted the occurrence of mitochondrial aggregation; at the same time, overexpression of *ARMC10* rescued mitochondrial fragments, reduced β‐amyloid (Aβ)‐induced neuronal apoptosis and exerted neuroprotective effects.^20^ This is consistent with our findings. From this, we concluded that ARMC10 protein is localized in the outer mitochondrial membrane, and overexpression of *ARMC10* can promote mitochondrial fusion and rescue OGD/R injury‐induced mitochondrial fragmentation and mitochondrial ultrastructure destruction, thereby protecting mitochondrial function and attenuating neuronal apoptosis.

The Wnt/β‐catenin signal pathway participates in the regulation of developmental processes and CNS mutation, including the proliferation, differentiation and migration of neuronal cells, axon growth and synaptogenesis, and plays an important role in inhibiting cell apoptosis and promoting cell survival. Liu et al. showed that enhancement of the Wnt/β‐catenin signalling pathway could protect hepatic mitochondrial function, reduce mitochondria‐mediated endogenous hepatocyte apoptosis and ultimately attenuate hepatic ischaemia/reperfusion injury.^34^ Mirra S et al found that *ARMC10* is expressed in chicken spinal cord nerve and participates in the regulation of chicken spinal cord nerve development by regulating Wnt/β‐catenin signal pathway.^35^ Therefore, we hypothesized that ARMC10 may be involved in cerebral ischaemia–reperfusion injury by regulating the Wnt/β‐catenin signalling pathway.

We examined the expression levels of key molecules (β‐catenin, GSK‐3β, p‐GSK‐3β) and downstream genes (*c‐Myc, CyclinD1, AXIN2*) of the Wnt/β‐catenin signalling pathway, and our results showed that overexpression of *ARMC10* activated the Wnt/β‐catenin signalling pathway, while knockdown of *ARMC10* could inhibit the activity of Wnt/β‐catenin signalling pathway. The transfer level of β‐catenin from cytoplasm to nucleus was crucial step that Wnt/β‐catenin signalling pathway plays an important role in biological functions. We found that knockdown of *ARMC10* significantly reduced the amount of β‐catenin protein in the nucleus, whereas overexpression of ARMC10 significantly increased the amount of β‐catenin protein in the nucleus, which further confirmed that *ARMC10* could activate the Wnt/β‐catenin signalling pathway. In addition, we further explored the relationship between the Wnt/β‐catenin signalling pathway and mitochondrial function and neuronal apoptosis using inhibitors and agonists of the β‐catenin signalling pathway, and the results showed that activation of the Wnt/β‐catenin signalling pathway could protect mitochondrial function and reduce neuronal apoptosis. Inhibition of the Wnt/β‐catenin signalling pathway can lead to mitochondrial dysfunction and promote neuronal apoptosis.

Finally, we constructed an OGD/R cell model using SH‐SY5Y cell line to further validate the role of *ARMC10*‐dependent Wnt/β‐catenin signalling pathway in brain I/R injury. We examined the expression levels of key molecules and downstream genes of the Wnt/β‐catenin signalling pathway, as well as mitochondrial function and neuronal apoptosis. The results showed that OGD/R treatment inhibited the activation of the Wnt/β‐catenin signalling pathway and decreased the expression of downstream target genes, while overexpression of *ARMC10* had a rescue effect. In addition, we found that OGD/R treatment induced a decrease in ATP production and an increase in ROS production, aggravating neuronal apoptosis. Overexpression of *ARMC10* rescued mitochondrial dysfunction induced by OGD/R injury, reduced neuronal apoptosis and exerted neuroprotective effects after IS. Interestingly, Wnt/β‐catenin signalling pathway agonists had similar mitochondrial protective effects compared with overexpression of *ARMC10*. Recent studies have shown that activation of the Wnt/β‐catenin signalling pathway improves neurological function by inhibiting mitochondrial oxidative stress, increasing mitochondrial membrane potential, inhibiting apoptosis and promoting mitochondrial biogenesis in Parkinson's disease rats.^36^ Consistent with previous studies, we also found that the protective effect of *ARMC10* against brain I/R injury was regulated by the Wnt/β‐catenin signalling pathway. Our results suggested that the Wnt/β‐catenin signalling pathway may serve as a promising candidate for neuroprotective therapy.

There are some limitations to this study. First, the clinical sample size is small and there may be some confounding factors, which may cause bias in the statistical results. It is necessary to further expand the sample size to investigate the changing pattern of *ARMC10* to provide data for clinical diagnosis and prognosis. Secondly, this study only investigated the mechanism by which *ARMC10* affects mitochondrial dynamics, mitochondrial function and neuronal apoptosis in ischaemic stroke in a cellular model, but lacked the corroboration of in vivo studies. The results of cellular experiments need to be further validated by constructing a mouse MCAO model.

## 5 CONCLUSION

In conclusion, our study demonstrates that *ARMC10* regulates mitochondrial dynamics and affects mitochondrial function through the Wnt/β‐catenin signalling pathway involved in cerebral ischaemia–reperfusion injury. Although accumulating evidence has revealed several proteins critical for the control of mitochondrial dynamics, such as Mfn1/2, Fis1, Mff and Drp1, novel targets that modulate this crucial biological process still urgently need to be identified. Our study reveals that *ARMC10* promotes mitochondrial fusion process to provide neuroprotection against ischaemia. Therefore, *ARMC10* is a therapeutic target for ischaemic stroke that acts by controlling mitochondrial homeostasis.

**Figure:**
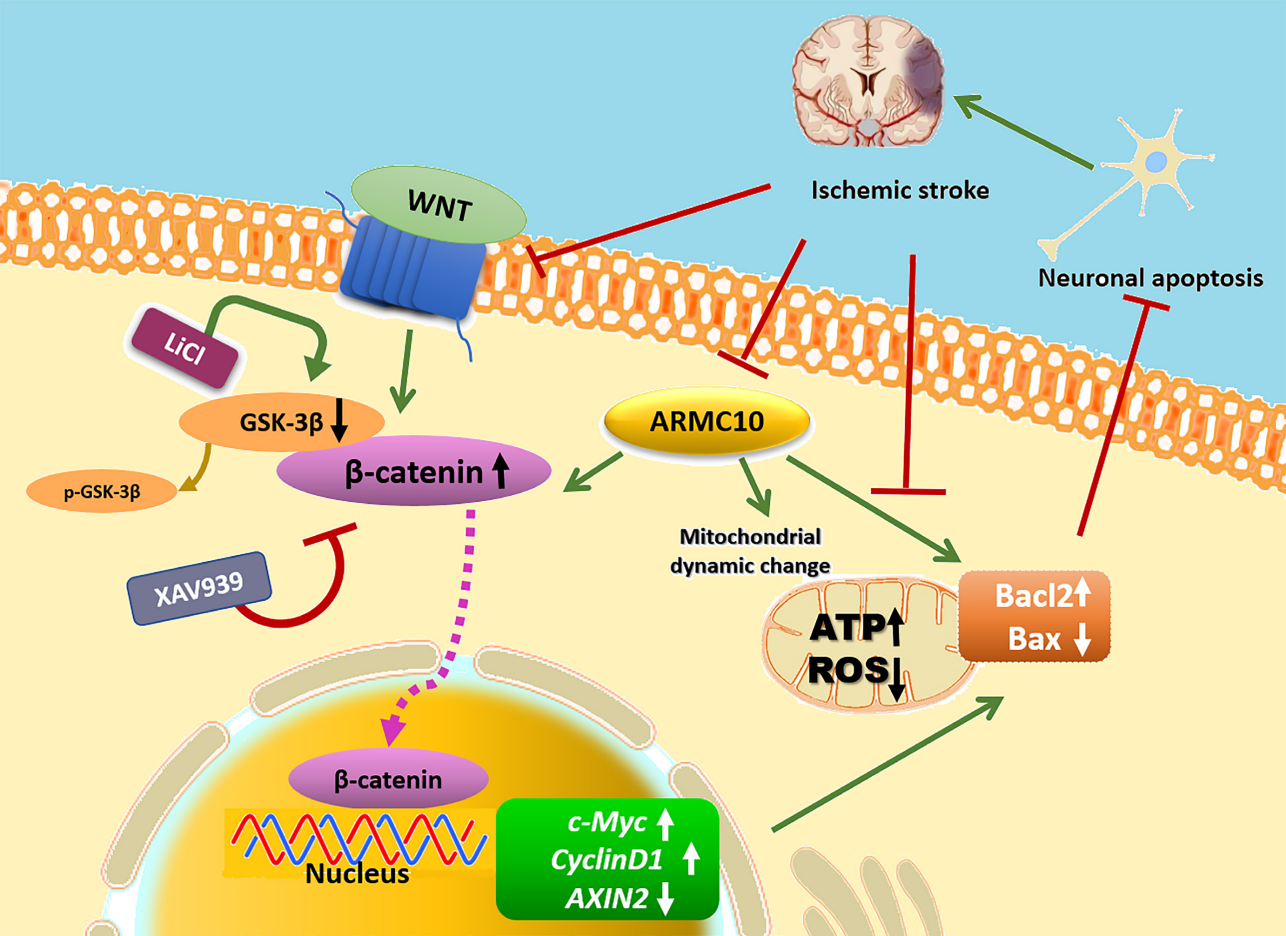
Working model.

## AUTHOR CONTRIBUTIONS

**Yanyang Huang:** Investigation (lead); validation (lead). **Zhaojing Zhang:** Investigation (equal); validation (equal). **Yatian Xu:** Resources (equal). **Yue Peng:** Resources (lead). **Ruochen Xu:** Resources (equal). **Yingying Luan:** Formal analysis (lead). **Xiaoshuai Bie:** Formal analysis (equal). **Jing Jia:** Conceptualization (equal); project administration (equal). **Chi Zhang:** Conceptualization (equal); project administration (equal). **Tianyi Han:** Formal analysis (equal). **Baixue Zhou:** Investigation (equal); validation (equal). **Zhihao Li:** Writing – original draft (equal). **Hong Zheng:** Conceptualization (equal); project administration (equal). **Dongzhi Yang:** Writing – original draft (lead). **Ying He:** Conceptualization (lead); project administration (lead).

## FUNDING INFORMATION

Research reported in this publication was supported by the National Natural Science Foundation of China (No. U2004114).

## CONFLICT OF INTEREST STATEMENT

The authors declare that they have no conflicts of interest with the contents of this article.

## Supporting information


Figures S1–S3.

## Data Availability

The data that support the findings of this study are available from the corresponding author upon reasonable request.
