# Supplementary material for: ARMC10 regulates mitochondrial dynamics and affects mitochondrial function via the Wnt/β‐catenin signalling pathway involved in ischaemic stroke
Source: J Cell Mol Med. 2024 Jun 25;28(12):e18449. doi: 10.1111/jcmm.18449 (PMC11196997; doi:10.1111/jcmm.18449)
Supplement: Supplementary file 1 — Figures S1–S3. [file JCMM-28-e18449-s001.docx]

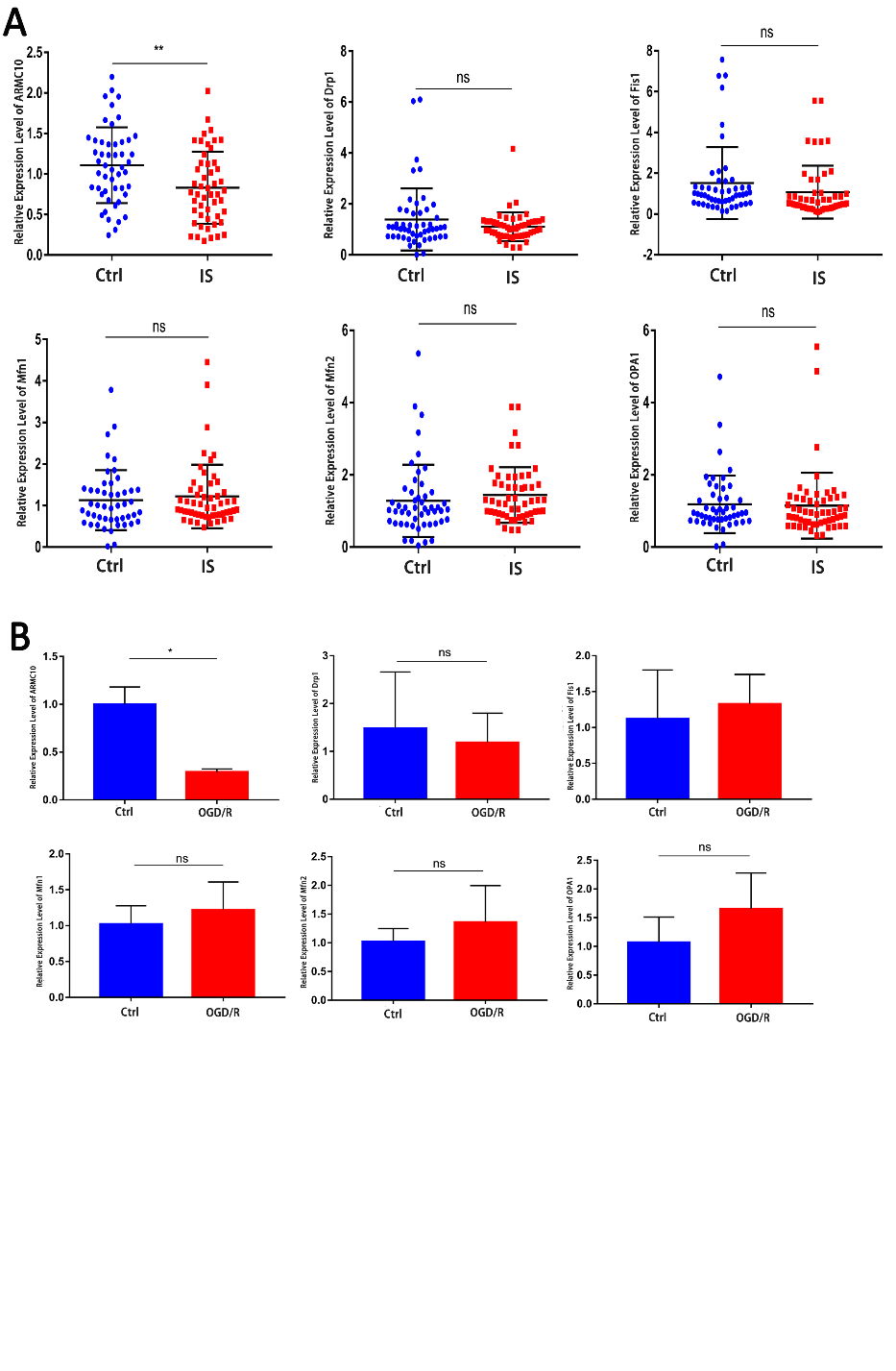
**Figure S1.** **Expression of genes related to mitochondrial dynamics.** (**P*<0.05, ***P*<0.01, ****P*<0.001) **A.** qPCR detect the expression of *ARMC10*，*Drp1*, *Fis1*, *Mfn1*, *Mfn2*, *OPA1* in peripheral blood of the control group and the IS group. **B**. qPCR detect *ARMC10*，*Drp1*, *Fis1*, *Mfn1*, *Mfn2*, *OPA1* expression levels of SH-SY5Y in control group and OGD/R group.


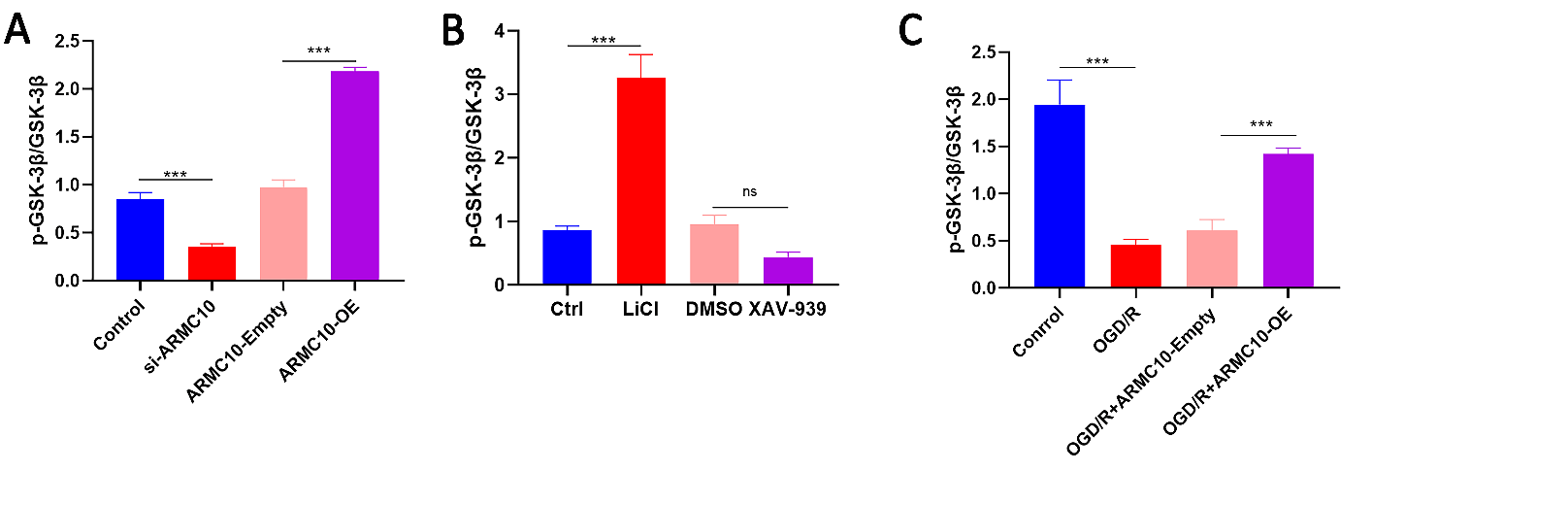
**Figure S2.** **Protein expression levels of p-GSK-3β.** (**P*<0.05, ***P*<0.01, ****P*<0.001) **A.** Protein expression level of p-GSK-3β after knock-down or overexpression of ARMC10. **B**. Protein expression level of p-GSK-3β after activating or inhibiting Wnt/ β-catenin pathway. **C**. Under the condition of OGD/R, the protein expression level of p-GSK-3β after knocking down or overexpressing ARMC10.


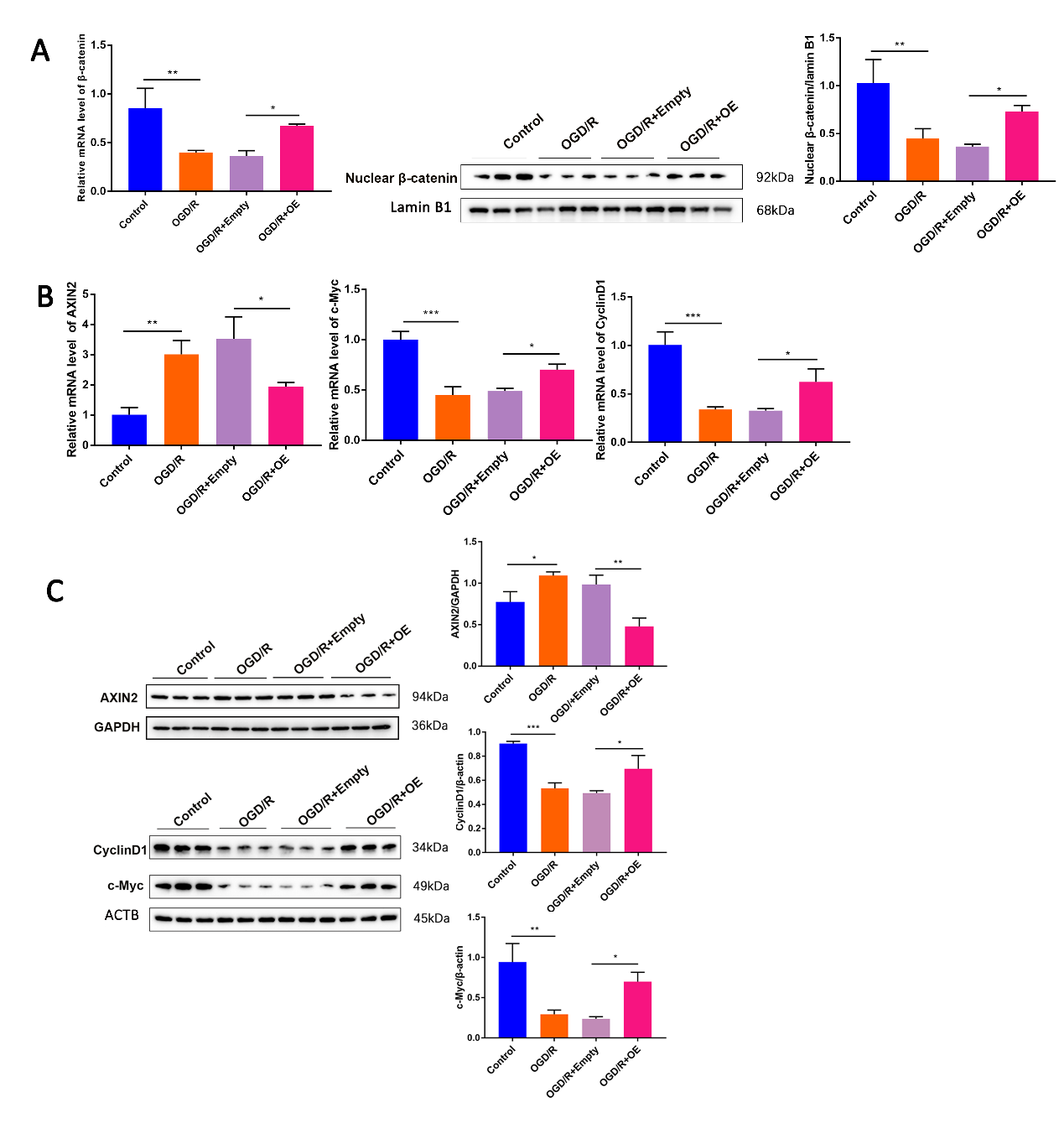


**Figure S3.** **ARMC10 regulates Wnt/ β-catenin signal pathway.** (**P*<0.05, ***P*<0.01, ****P*<0.001) **A.** qPCR and WB was used to detect expression levels of β-catenin. **B**. qPCR detected expression of downstream target genes of Wnt/β-catenin signaling pathway. **C**. Western Blot determined the expression of downstream target genes of Wnt/β-catenin signaling pathway.（S3C and 7A、7C share the same batch of GAPDH bands in their WB results.）
